# The Role of *miR-217-5p* in the Puromycin Aminonucleoside-Induced Morphological Change of Podocytes

**DOI:** 10.3390/ncrna8030043

**Published:** 2022-06-08

**Authors:** Osamu Ishibashi, Mika Hayashi, Aya Horikawa, Hitoshi Owada, Ryotaro Miyamoto, Naoya Mizukami, Takashi Inui

**Affiliations:** Laboratory of Biological Macromolecules, Graduate School of Agriculture, Osaka Metropolitan University, Sakai 599-8531, Japan; hymika0125@gmail.com (M.H.); holy0w0@gmail.com (A.H.); d224898@hiroshima-u.ac.jp (H.O.); sc22894c@st.omu.ac.jp (R.M.); pascal1813@icloud.com (N.M.); inuit@omu.ac.jp (T.I.)

**Keywords:** micoRNA, podocyte, puromycin aminonucleoside, RNA-seq, actin cytoskeleton

## Abstract

Podocytes, alternatively called glomerular epithelial cells, are terminally differentiated cells that wrap around glomerular capillaries and function as a part of the glomerular filtration barrier in the kidney. Therefore, podocyte injury with morphological alteration and detachment from glomerular capillaries leads to severe proteinuria and subsequent renal failure through glomerulosclerosis. Previous RNA sequencing analysis of primary rat podocytes exposed to puromycin aminonucleoside (PAN), a well-known experimental model of injured podocytes, identified several transcripts as being aberrantly expressed. However, how the expression of these transcripts is regulated remains unclear. MicroRNAs (miRNAs) are small noncoding RNAs that posttranscriptionally inhibit the expression of their target transcripts. In this study, using small RNA sequencing analysis, *miR-217-5p* was identified as the most upregulated transcript in PAN-treated rat podocytes. *MiR-217-5p* overexpression in E11 podocyte cells led to shrunken cells with abnormal actin cytoskeletons. Consistent with these changes in cell morphology, gene ontology (GO) enrichment analysis showed that interactive GO terms related to cell morphogenesis were enriched with the predicted targets of *miR-217-5p*. Of the predicted targets highly downregulated by PAN, *Myosin 1d* (*Myo1d*) is a nonmuscle myosin predicted to be involved in actin filament organization and thought to play a role in podocyte morphogenesis and injury. We demonstrated that *miR-217-5p* targets Myo1d by luciferase assays, qRT–PCR, and Western blotting. Furthermore, we showed that *miR-217-5p* was present in urine from PAN- but not saline-administrated rats. Taken together, our data suggest that *miR-217-5p* may serve as a therapeutic target and a biomarker for podocyte injury.

## 1. Introduction

Approximately 10% of the world population is affected by chronic kidney disease (CKD), defined as abnormalities in kidney structure and/or function that are present for at least 3 months, irrespective of cause [1]. Millions of patients with CKD die every year due to inadequate treatment [1]. CKD shows few symptoms in initial stages and is often detected in advanced stages when symptoms become more obvious [2,3]. For this reason, CKD is often classified as a “silent” disease. When kidney functions are practically lost, this is expressed as ‘renal failure’ [4,5]. Unfortunately, no therapeutic methods to restore the function of chronically impaired kidneys have been established. During late stages of CKD, patients with CKD require renal replacement therapies including hemodialysis and kidney transplants for survival. Although hemodialysis is generally applied in cases of renal failure, patients receiving dialysis have significantly decreased life expectancy compared to the normal population [6]. Hemodialysis also reduces patient quality of life in physiological, mental, and/or social aspect(s) [7]. Furthermore, the high cost of dialysis places an economic burden on patients and is suggested to exert a negative influence on health economics [7]. While kidney transplantation can be a therapy option for end-stage CKD patients [8,9], the outcome is controversial among health care professionals [10,11]. Furthermore, patients usually need to wait a long time for a compatible donor and may die while waiting for a kidney [12]. Finally, for a transplant to be considered successful, the donated kidney must be accepted by the patient’s immune system [13].

In the kidney, glomeruli are found in the cortex and serve as a hemofiltration device [14]. Podocytes, alternatively called glomerular epithelial cells, are highly differentiated cells that cover glomerular capillaries. Podocytes, together with endothelial cells and glomerular basal membranes, form a unit that functions as a glomerular filtration barrier in the kidney [15,16]. Podocytes have a characteristic morphology that contains many foot processes [14]. Between the foot processes, membranous structures called slit membranes form, and these membranes play an essential role as a final filtration barrier to prevent leakage of proteins into the urine during glomerular filtration [14,15,16]. The structure of the filtration barrier can be disrupted by podocyte damage, which occasionally causes podocyte detachment from the glomeruli; in these cases, severe proteinuria leading to renal failure may occur [16,17]. However, the mechanisms that underlie the structural disruption of podocyte remain unclear.

MicroRNAs (miRNAs) are small noncoding RNAs of 19–25 nucleotides in length that play a crucial role in posttranscriptional gene regulation by repressing the translation of or degrading target messenger RNAs (mRNA) [18,19]. Notably, mice with podocyte-specific deficiency in Dicer, a nuclease essential for miRNA biosynthesis, have been shown to develop severe proteinuria and die within weeks, indicating that miRNAs are indispensable for podocyte function [20,21,22]. Previous studies have also demonstrated that many miRNAs play essential roles in nephropathy-related podocyte injury [23,24]. However, the mechanisms underlying miRNA-mediated podocyte injury are not fully understood. Here, we performed an integrative analysis of miRNA and mRNA expression profiles associated with puromycin nucleoside (PAN)-induced podocyte injury. Our analysis showed that *miR-217-5p* upregulation in PAN-treated podocytes can lead to morphological alteration. Furthermore, we demonstrated that *miR-217-5p* is present in urine from PAN- but not saline-administrated rats, suggesting that *miR-217-5p* may serve as a therapeutic target and biomarker for podocyte injury.

## 2. Materials and Methods

### 2.1. Cell Culture and Induction of Podocyte Injury

Podocytes in primary culture were isolated from male Wistar rats at ages 7–8 weeks (SLC, Hamamatsu, Japan) and cultured as described previously [25,26]. The mouse podocyte cell line E11 was purchased from Cell Lines Service GmbH (Eppelheim, Germany) and cultured as described previously [27]. To cause cell injury, primary rat podocytes and E11 cells were treated with various concentrations of PAN (Wako, Osaka, Japan) for 24 h or 48 h.

### 2.2. Comprehensive Small RNA Sequencing

One μg of total RNA isolated from the rat podocytes in primary culture was subjected to small RNA sequencing to comprehensively analyze miRNA expression. Small RNA libraries were constructed using the TruSeq Small RNA Library Preparation Kits (Illumina, San Diego, CA, USA) and analyzed on a HiSeq-3000 sequencer (Illumina) at the Genome Information Research Centre of Osaka University. Raw reads obtained from the sequencing analysis were trimmed using Cutadapt v1.9.2 and subjected to miRNA-derived read counting and annotation using CLC Genomics Workbench 11.0.1 (Qiagen, Venlo, The Netherlands).

The reads obtained in this analysis and related metadata were deposited in the DNA Data Bank of Japan Sequence Read Archive (DRA) under the accession number DRA013173.

### 2.3. Real-Time Reverse-Transcription PCR for miRNA and mRNA

Quantitative RT-PCR (qRT–PCR) analysis using TaqMan^®^ MicroRNA Assays (Thermo-Fisher Scientific, Waltham, MA, USA) was conducted to validate the results of miRNA-seq analysis. Because expression levels of target miRNAs *miR-217-5p* were low, PCR preamplification with 12 cycles was conducted using TaqMan^®^ PreAmp Master Mix (Thermo-Fisher Scientific) before performing qRT–PCR. The expression of *U6* small nuclear RNA (snRNA) was analyzed as an endogenous internal control to normalize expression levels of miRNAs. Monitoring the amplification of PCR products was conducted on a DICE Thermal Cycler Real-Time System (Takara-bio, Kusatsu, Japan).

Thunderbird SYBR qPCR Mix (Toyobo, Osaka, Japan) was used for the quantitative analysis of mRNA expression levels. *GAPDH* mRNA or *18S rRNA* was used as an endogenous internal control to normalize mRNAs’ expression levels (the primers sequences are listed in Table S1). Monitoring of amplification of PCR products was conducted on a DICE Thermal Cycler Real-Time System (Takara-bio). Dissociation curve analyses were also performed to verify specific amplification.

### 2.4. miRNA Overexpression in E11 Podocytes

To overexpress *miR-217-5p* in E11 podocytes, the cells were transfected with mirVana miRNA Mimic-*miR-217-5p* (Thermo-Ficsher Scientific) at 50 nM using Lipofectamin RNAiMAX (Thermo-Ficsher Scientific). As a control, mirVana miRNA Mimic-Negative Control #1 (Thermo-Ficsher Scientific) was also used for transfection.

### 2.5. Cell Morphology Observation and Fluorescent Immunocytochemistry

Cell morphology in culture was routinely observed using an Olympus CKX41 inverted microscope (Olympus, Tokyo, Japan). For immunocytochemistry, cells were cultured on tissue culture-treated chamber slides, fixed with 10% formalin in PBS, permeabilized with 0.2% Triton-X100 in PBS, blocked with 1% bovine serum albumin (BSA) in PBS, and reacted with the rabbit anti-β-actin antibody (PM053-7) (MBL, Tokyo, Japan) followed by the Alexa488-labeled antirabbit IgG antibody (ab150077) (Abcam, Cambridge, UK). Fluorescent cells were observed under a BZ9000 fluorescence microscope (Keyence, Osaka, Japan).

### 2.6. Cell Viability Assay

E11 cells were seeded in 96-well plates at 2 × 10^3^ cells/well, cultured overnight, and treated with PAN (Wako, Osaka, Japan) for 48 or 96 h. After changing to fresh medium, the viability of podocytes was evaluated using Cell Counting Reagent SF (Nacalai Tesque, Kyoto, Japan) according to the manufacturer’s instructions.

### 2.7. Western Blotting

Cells were lysed with a RIPA buffer (50 mM Tris-HCl, pH 7.4; 150 mM NaCl; 1% NP-40; 0.5% sodium deoxycholate). Cell lysates were subjected to 8% SDS-polyacrylamide gel electrophoresis and transferred to Immobilon polyvinylidene difluoride membranes (Merck, Kenilworth, NJ, USA). The blots were then probed with the mouse anti-Myo1d monoclonal antibody (sc-515292) (Santa Cruz, Dallas, TX, USA) followed by the goat antimouse IgG antibody linked with horseradish peroxidase (HRP) (#330) (MBL). The same blots were also probed for β-actin using the HRP-linked rabbit anti-β-actin antibody (PM053-7) (MBL) as an internal control. Immobilon ECL Ultra Western HRP Substrate and Immobilon Western HRP Substrate (Merck-Millipore, Burlington, VT, USA) were used to detect Myo1d and β-actin, respectively. A LAS-4000 lumino-image analyzer (Thermo-Fisher Scientific) was used to detect chemiluminescent signals.

### 2.8. Reporter Plasmid Construction and Luciferase Assay

The 2.2-kbp 3′-UTR of mouse *Myo1d* mRNA was amplified by RT-PCR from the total RNA of E11 cells using PrimeScript II reverse transcriptase (Takara-bio) and KOD-Plus DNA polymerase (Toyobo). The primers used for RT-PCR amplification were: 5′-CTGCTGCACATCAGAGGCCT-3′ (forward) and 5′-TTTGTCGACAAGATTTAATGCTTTATTGC-3′ (reverse). The PCR product encompassing the 3′-UTRs of *Myo1d* was then subcloned into the pmirGLO vector (Promega, Madison, USA) at the DraI and SalI restriction sites to construct the pmirGLO-*Myo1d* 3′-UTR luciferase reporter plasmid. DNA ligation was conducted at 16 °C for 30 min using DNA Ligation Kit Ver.1 (Takara-bio). A reporter plasmid having a mutation at the predicted *miR-217-5p* binding site in the 3′-UTR of *Myo1d* mRNA was constructed using pmirGLO-*Myo1d* 3′-UTR as a template. First, the part between the predicted *miR-217-5p* binding site and the end of the 3′-UTR was PCR-amplified using KOD-Plus DNA polymerase (Toyobo) and the following primers: 5′-ACCTGGAATTCGGGGTGTGACTGACCACAGTAACAGCAGAGGAGAGGACACAGTGATTGTATGCATGGAGTAGGGGTCTCTTGAGTTAATGAAGATATCGTTATGGTTTG-3′ (forward) and 5′-TTTGTCGACAAGATTTAATGCTTTATTGC-3′ (reverse). The generated amplicon was digested using EcoRI and SalI at both termini. The amplicon then substituted the corresponding region of pmirGLO-*Myo1d* 3′-UTR. For reporter assays, E11 cells were transfected with these plasmids together with synthetic miRNA mimics (Thermo-Fisher Scientific) at 20 nM using Lipofectamin2000 (Thermo-Fisher Scientific). Twenty-four hours after transfection, the cells were processed using the Dual-Luciferase Reporter Assay System (Promega). Luminescence was detected using a TD-20/20 luminometer (Promega).

### 2.9. Urine Processing

PAN administration in Wistar rats and urine collection from rats were performed as described previously [25]. Urine samples were collected for 12 h at day 5 following the administration of PAN or saline. Collection on day 5 was chosen because we previously showed that urinary protein concentration, an index to evaluate the progression of glomerulopathy, was markedly increased from day 5 onwards [25]. The collected urine was filtered through a 0.22-μm filter to remove debris, then subjected to RNA isolation using an RNAiso Blood reagent (Takara, Tokyo, Japan). In parallel, to monitor the progression of glomerulopathy, the protein concentration of the filtered urine was measured as described previously [25].

### 2.10. Bioinformatics

mRNAs meeting the following criteria were selected as PAN-regulated mRNAs: (i) FPKM value of podocytes cultured with PAN >10, (ii) FPKM ratio (podocytes cultured with PAN/podocytes cultured without PAN) >1.2 or <0.8, and (iii) the false discovery rate (q-value) < 0.05. Putative miRNA targets were predicted using TargetScan v7.2 (http://www.targetscan.org/vert_72/, accessed on 6 June 2022) as described previously [28]. Enriched GO terms and interactive ontology corresponding to a specific gene were retrieved using GOnet [29], which predict biological processes associated with the predicted targets of *miR-217-5p* in podocytes.

### 2.11. Statistical Analysis

Student’s *t*-tests were used to assess the significance of differences among groups for comparisons between two groups, and one-way analysis of variance followed by Ryan’s test was used for comparisons among three or more groups. In all analyses, *p* < 0.05 was considered to be statistically significant.

## 3. Results

### 3.1. Identification of miRNAs Dysregulated by PAN-Induced Podocyte Injury

**Figure 1:**
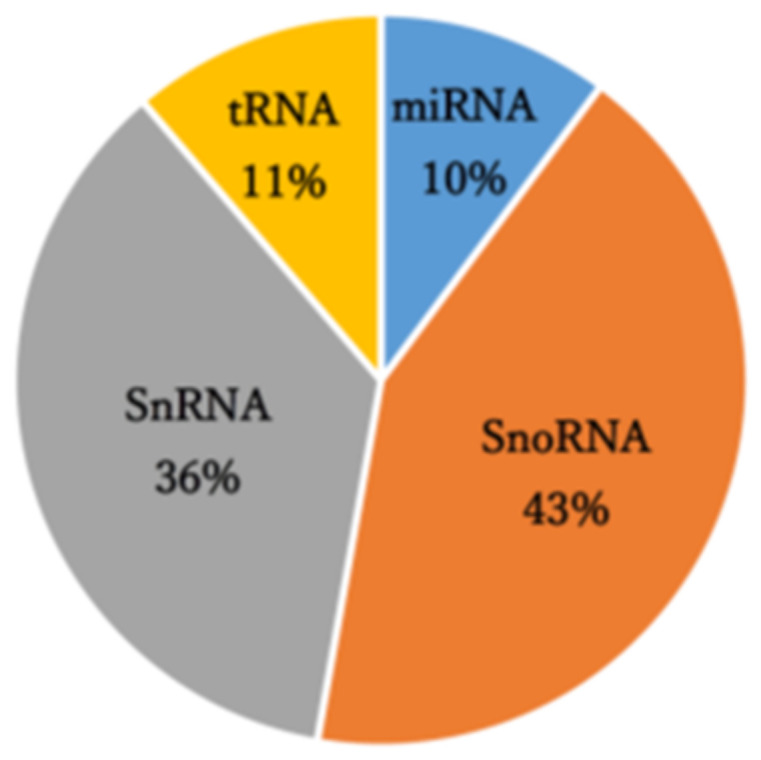
Small RNA class composition of the small RNA sequencing-derived reads.

A small RNA sequencing analysis of control and PAN-treated primary rat podocytes that manufactured 8.64–13.5 million raw reads was performed. The mapping results of this analysis are summarized in Table S2. Reads obtained through small RNA-seq analysis were mapped to 3,948 annotated small RNAs comprising small nucleolar RNA (43%), small nuclear RNA (36%), transfer RNA-derived small RNAs (11%), and miRNAs (10%) (Figure 1). In the analysis of miRNA-derived reads, *miR-217-5p*, *-216a-5p*, *-338-3p*, and *-3583-5p* were identified as miRNAs with significantly enhanced expression (over 2-fold) following PAN-induced podocyte injury (Table 1). Conversely, *miR-3572* expression was significantly reduced in response to PAN-induced podocyte injury (Table 1).

### 3.2. Validation of PAN-Induced miR-217-5p Expression in Primary Rat Podocytes

**Figure 2:**
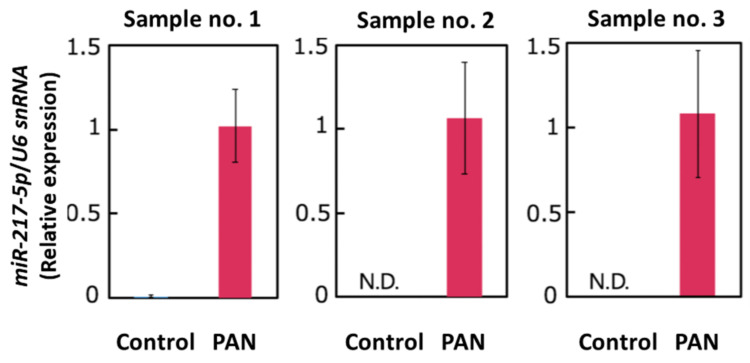
Expression of *miR-217-5p* in PAN-treated rat podocytes. *MiR-217-5p* expression level was normalized relative to *U6 snRNA* expression. The normalized expression level of *miR-217-5p* at 3 μg/mL PAN was given an arbitrary value of 1. Data represent the means ± SD (n = 3).

Our small RNA-seq analysis indicated that *miR-217-5p* is the most highly (5.7-fold) upregulated miRNA in PAN-treated podocytes; no other miRNAs were upregulated more than five times. We performed qRT–PCR analysis to verify PAN-induced *miR-217-5p* expression in primary rat podocytes. Analysis of qRT–PCR analysis revealed that *miR-217-5p* expression in rat podocytes was markedly increased following PAN treatment (Figure 2).

### 3.3. PAN-Induced Effects on Cell Viability, Cell Morphology, and miR-217-5p Expression in E11 Podocyte Cells

**Figure 3:**
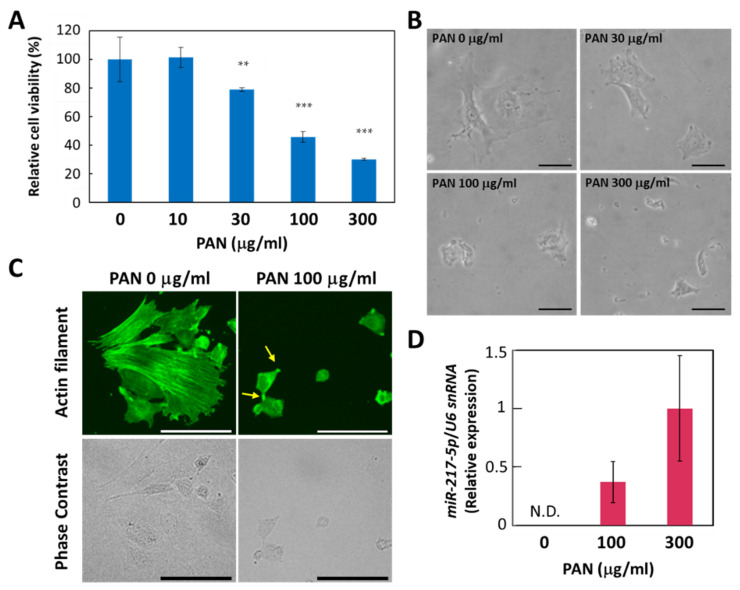
PAN-induced effects on cell viability, cell morphology, and *miR-217-5p* expression in E11 podocytic cells. (**A**) PAN-induced reduction in the cell viability of E11 cells. Cell viability after PAN (0–300 μg/mL) exposure for 48 h was evaluated by WST-8 assay. Cell viability at 0 μg/mL PAN was given an arbitrary value of 100%. ** *p* < 0.01 versus 0 μg/mL PAN. *** *p* < 0.001 versus 0 μg/mL PAN. Data represent the means ± SD (n = 3). (**B**) Phase contrast images of E11 cells in culture after PAN (0–300 μg/mL) exposure for 48 h. Bars: 50 μm. (**C**) Actin cytoskeletons of E11 cells treated with or without PAN (100 μg/mL) for 48 h were visualized by immunostaining with anti-β-actin antibody (top). Phase contrast images of the corresponding cells are also shown (bottom). Representative processlike structures appearing in some PAN-treated cells are indicated by arrows. Bars: 100 μm. (**D**) Expression of *miR-217-5p* in PAN-treated E11 cells. *MiR-217-5p* expression in E11 cells treated with PAN (0–300 mg/mL) was normalized relative to *U6 snRNA* expression. The relative expression level of *miR-217-5p* at 300 μg/mL PAN was given an arbitrary value of 1. Data represent the means ± SD (n = 3). N.D. not detected.

Considering the drastic induction of *miR-217-5p* expression in rat podocytes, we sought to determine whether the increased expression of *miR-217-5p* was involved in PAN-induced podocyte injury. However, primary rat podocytes are not suitable for this purpose because only a small number of podocytes can be isolated from a rat. Furthermore, it is generally difficult to efficiently transfect primary cells with DNA or RNA. Therefore, we evaluated whether E11, an immortalized cell line generated from mouse podocytes, could be used as an alternative in vitro model, since siRNA-mediated gene knockdown and vector-mediated gene overexpression have been previously conducted in this cell line [30]. First, we evaluated the effect of PAN on E11 cell viability. We found that PAN exerted a strong suppressive effect on E11 cell viability at >100 μg/mL (Figure 3A). This concentration is higher than effective PAN concentrations reported previously for primary rat podocytes (>10 μg/mL) [25]. The PAN-resistant properties of mouse podocytes compared to rat podocytes has been attributed to a deficiency in the adenosine deamination pathway [31] and low expression of plasma membrane amine transporter [32].

We also found that PAN induced morphological changes in E11 cells. While E11 cells cultured in the absence of PAN largely exhibit a spread-out shape, cells appear shrunken when cultured with 100 μg/mL PAN (Figure 3B). In addition, immunostaining with an anti-β-actin antibody revealed that actin filaments lining appeared disorganized or shortened in E11 cells treated with 100 μg/mL PAN (Figure 3C). 

Next, we evaluated whether *miR-217-5p* expression is upregulated in PAN-treated E11 cells. We found that miR-217-5p expression was upregulated in E11 cells treated with 100 and 300 μg/mL PAN (Figure 3D). These data suggest that PAN exerts similar effects on cell viability and miR-217-5p expression in both primary rat podocytes and E11 cells, indicating that E11 cells can be used as a model to investigate the role of miR-217-5p in PAN-induced podocyte injury.

### 3.4. MiR-217-5p-Induced Effects on Cell Viability and Cell Morphology in E11 Cells

**Figure 4:**
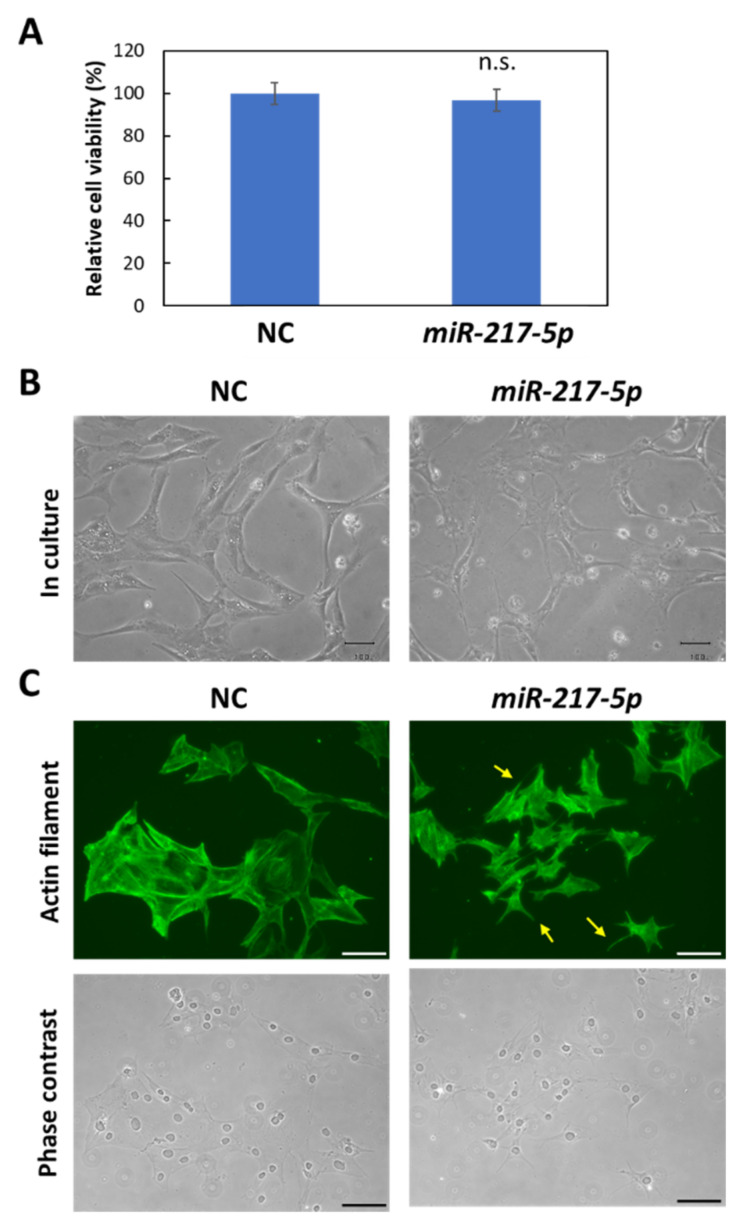
Morphological abnormalities in *miR-217-5p*-transfected cells. (**A**) Effects of induced *miR-217-5p* expression in E11 cells on cell viability. Cell viability at day 2 following the transfection was evaluated. n.s.: not significant compared to the negative control miRNA mimic (NC)-transfected cells. (**B**) Phase contrast images of E11 cells transfected with the negative control miRNA (NC) or *miR-217-5p* mimics at 48 h posttransfection. Bars: 50 μm. (**C**) Actin cytoskeleton of E11 cells transfected with NC or *miR-217-5p* mimics at 48 h posttransfection. Actin filaments were visualized by immunostaining with anti-β-actin antibody (top). Processlike structures in the *miR-217-5p*-transfected cells are indicated by arrows. Phase contrast images of cells are also shown (bottom). Bars: 100 μm.

We next evaluated whether *miR-217-5p* overexpression in E11 cells affects cell viability and morphology. *MiR-217-5p* overexpression did not significantly affect cell viability of E11 cells after 48 h (Figure 4A) and 96 h (Figure S1). However, E11 cells with *miR-217-5p* overexpression in culture exhibited shrunken shapes (Figure 4B). Furthermore, immunostaining with an anti-β-actin antibody revealed that actin filaments lining behind the cell membrane appeared disorganized or shortened in E11 cells with *miR-217-5p* overexpression (Figure 4C). These data imply that *miR-217-5p* is possibly involved in cell morphogenesis through the regulation of actin filament formation.

### 3.5. Integrative Analysis of miRNA and mRNA Expression in PAN-Treated Podocytes

Although miRNAs are known to silence their target genes by attenuating translation and degrading mRNAs, previous studies have shown that mammalian miRNAs predominantly reduce target mRNA levels [33,34]. Therefore, expression levels of PAN-regulated miRNAs and their target mRNAs in podocytes are thought to be reciprocally regulated. In the present study, we selected PAN-regulated mRNAs as described in the Materials and Methods section. Through screening based on these criteria, 226 and 480 mRNAs were identified as upregulated and downregulated mRNAs, respectively (Figure S2, Tables S3 and S4). Next, the PAN-regulated mRNAs were screened to identify *miR-217-5p* targets satisfying the following criteria: (i) expression inversely correlated with *miR-217-5p* and (ii) in silico-predicted targets of *miR-217-5p*. After proceeding with these procedures, 12 mRNAs were identified as the predicted targets of *miR-217-5p* (Figure S3 and Table 2).

### 3.6. Gene Ontologies Associated with the Predicted Targets of the PAN-Dysregulated miRNAs

GO enrichment analyses were performed using GOnet to predict biological processes associated with the predicted targets of *miR-217-5p* in podocytes (Table 2). Consistent with the *mR-217-5p*-induced abnormal morphology of E11 cells, interactive GO (biological process) terms including those related to cell morphogenesis were enriched with the predicted targets of *miR-217-5p* (Figure S4). This study focused on *Myosin 1d* (*Myo1d*) as a predicted target because it is annotated to GO terms related to actin filament organization (Table 2), and previous studies have shown the involvement of nonmuscle myosins in podocyte morphogenesis and injury [35].

**Table 2:** Predicted target mRNAs of *miR-217-5p* in PAN-treated podocytes and GO annotations.

mRNA	Fold Change	Representative GO (Biological Process) Annotations
*Pard3b*	0.303	cell adhesion, cell cycle, cell division, etc.
*Fndc3b*	0.407	integral component of membrane
*Myo1d*	0.430	actin filament organization, cellular localization, etc.
*Akap2*	0.493	actin filament organization, protein localization, etc.
*Peak1*	0.532	cell migration, focal adhesion assembly, etc.
*Srgap1*	0.551	negative regulation of cell migration, etc.
*Fn1*	0.573	acute-phase response, acute-phase response, etc.
*Picalm*	0.604	amyloid-beta clearance by transcytosis, axonogenesis, etc.
*Meis1*	0.610	Angiogenesis, animal organ morphogenesis, etc.
*Epha7*	0.620	Angiogenesis, animal organ morphogenesis, etc.
*Crls1*	0.716	lens morphogenesis in camera-type eye, etc.
*Rock1*	0.733	regulation of cell population proliferation, etc.

**Figure 5:**
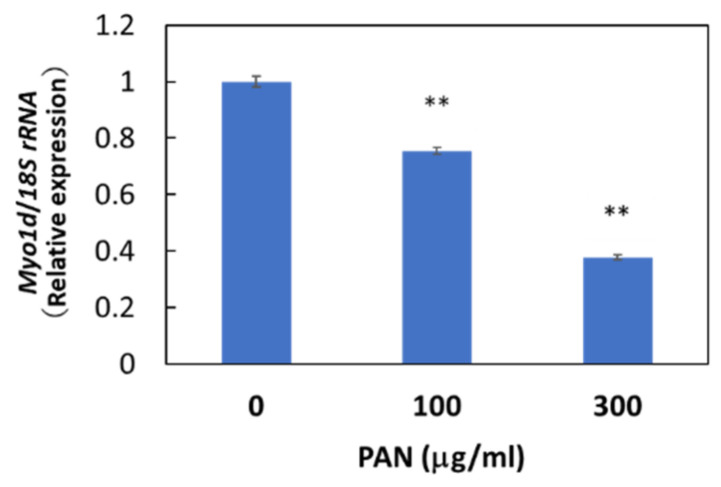
*Myo1d* mRNA levels in PAN-treated E11 cells. E11 cells were treated with 0–300 μg/mL PAN. Values were normalized relative to *18S* rRNA expression. The relative expression level of *Myo1d* mRNA at 0 μg/mL PAN was given arbitrary value of 1. ** *p* < 0.01 versus 0 μg/mL PAN. Data represent the means ± SD (n = 3).

Immunohistochemical staining showed the cytosolic localization of Myo1d protein in E11 cells, consistent with the previously reported localization of Myo1d protein in the human bone osteosarcoma cell line U-2 OS (Figure S5). We then examined whether *Myo1d* mRNA expression in podocytes was downregulated by PAN as shown in our microarray analysis. As expected, PAN downregulated *Myo1d* mRNA expression levels in primary rat podocytes (Figure S6) and E11 cells (Figure 5), and PAN upregulated *miR-217-5p* expression in these cells (Figure 3D). The reciprocal regulation of *miR-217-5p* and *Myo1d* mRNA expression raises the possibility that the *miR-217-5p*-Myo1d axis may be involved in PAN-induced podocyte injury.

This possibility was supported by analyses of publicly available datasets of podocyte injury-related transcriptomes. The analysis of the dataset GSE124622, created through microarray analysis of immortalized human podocytes treated with PAN and Adriamycin (ADR) to cause podocyte injury [36], showed that *Myo1d* mRNA levels in the PAN- and ADR-treated podocytes decreased 0.41-fold (*p* = 7.54E-06) and 0.64-fold (*p* = 0.0364) compared to control podocytes, respectively (Table S5). Furthermore, analysis of the dataset GSE108629 analysis, created through a microarray-based study using a mouse model of immunotoxin-inducible podocyte injury, showed that *Myo1d* mRNA levels were reduced 0.61-fold (*p* = 0.00158) after podocyte injury [37]. These results indicate that a reduction in *Myo1d* mRNA expression in injured podocytes was observed in our rat model and in in vitro or in vivo models from other organisms.

### 3.7. Target Validation of miR-217-5p

**Figure 6:**
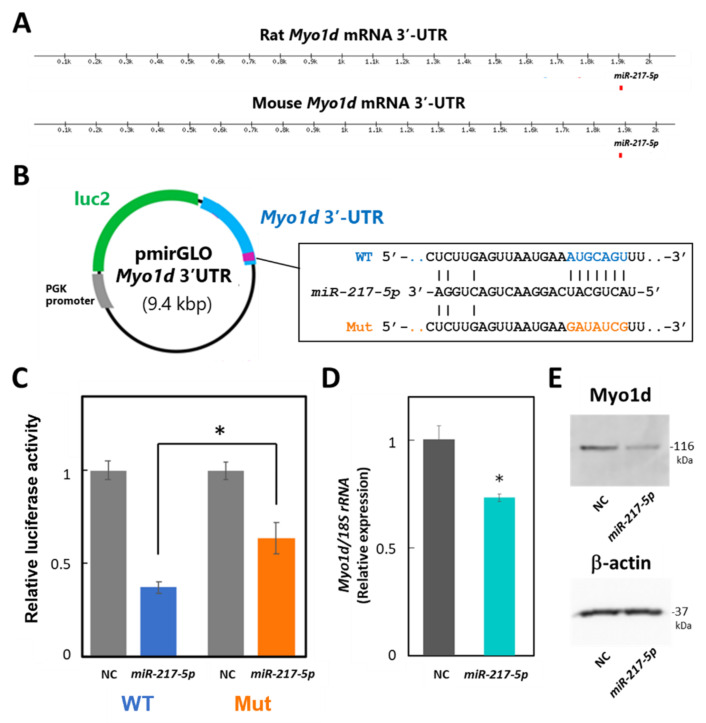
Target validation of *miR-217-5p* in PAN-treated rat podocytes. (**A**) Schematic illustration showing the putative position of *miR-217-5p* binding sites in the 3′-UTRs of mouse and rat *Myo1d* mRNAs predicted by TargetScan Human 8.0. (**B**) Schematic illustration showing the structure of the reporter plasmid with the wild-type (WT) and mutated (mut) 3′-UTR of mouse *Myo1d*. (**C**) A luciferase reporter assay examining targeting of the *Myo1d* 3′-UTR by *rno-miR-217-5p*. The relative light units (RLUs) of firefly luciferase activity under the control of the *Myo1d* 3′-UTR were normalized to those of constitutively controlled Renilla luciferase. The average relative RLUs of luciferase activity in the negative control miRNA mimic (NC)-transfected cells was set at 1. Data represent the means ± SD of triplicate measurements. * *p* < 0.05 versus control. (**D**) Expression levels of the *Myo1d* mRNA in E11 cells transfected with the *miR-217-5p* mimic or NC. *Myo1d* mRNA levels were analyzed using qRT-PCR, and the data were normalized to the expression level of *18S* rRNA. The average normalized *Myo1d* mRNA expression level in NC-transfected cells was set at 1. (**E**) Expression levels of the Myo1d protein in E11 cells transfected with the *miR-217-5p* mimic or NC. Myo1d protein expression was analyzed using Western blotting. The expression of β-actin protein as an internal control was analyzed on the same blot. The original images of blots from two independent experiments are shown in Figure S7.

To evaluate whether *miR-217-5p* targets *Myo1d* mRNA in podocytes through its 3′-UTR, we performed luciferase assays in E11 cells. Because the 3′-UTRs of mouse and rat *Myo1d* mRNAs are highly similar (84%) and have a predicted common *miR-217-5p* binding site at identical positions of their 3′-UTRs (Figure 6A), we constructed reporter plasmid containing the mouse *Myo1d* 3′-UTR, designated as pmirGLO-*Myo1d*-3′-UTR (Figure 6B). Cotransfection of *miR-217-5p* showed significantly downregulated luciferase activity in the reporter plasmid-transfected cells compared to the control (Figure 6C). Alternatively, when E11 cells were transfected with the reporter plasmid with a mutation at the predicted *miR-217-5p*-binding site in the *Myo1d* 3′-UTR (Figure 6B), the inhibitory effect of *miR-217-5p* on luciferase activity was weakened (Figure 6C). However, the mutation only partly restored the luciferase activity attenuated by *miR-217-5p*. Therefore, there may be other *miR-217-5p* recognition site(s) in the 3′-UTR of *Myo1d* mRNA, which were not identified by the target prediction tool.

Next, we evaluated whether *miR-217-5p* overexpression in E11 cells affects Myo1d mRNA and protein expression levels through qRT–PCR and Western blotting, respectively. As expected, expression levels of both *Myo1d* mRNA and Myo1d protein were reduced by overexpression of *miR-217-5p* compared to the control (Figure 6D,E).

### 3.8. Detection of miR-217-5p in Urine from PAN-Administrated Rats

**Figure 7:**
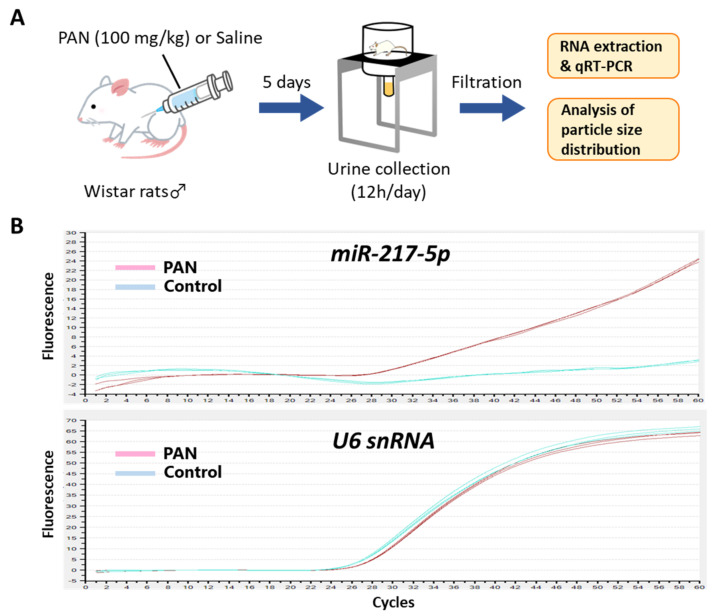
Detection of *miR-217-5p* in urine from PAN-administrated rats. (**A**) Schematic illustration of the experimental procedure. Seven-week-old male Wistar rats were administrated with 100 mg/kg PAN or saline. (**B**) Amplification plots of qRT–PCR for the detection of *miR-217-5p* and *U6 snRNA* in urine from the PAN- or saline-administrated rats. Urine samples collected at day 5 following PAN/saline administration were used for the analysis.

Because podocytes face the urinary space of the Bowman’s capsule, it is conceivable that miRNAs in podocytes are secreted into glomerular filtrate. We evaluated the amount of *miR-217-5p* in urine from PAN- and saline-administrated rats (Figure 7A). Consistent with the marked increase of *miR-217-5p* expression in PAN-treated rat podocytes (Figure 2), *miR-217-5p* was detected in cell-free urine from PAN-administrated rats but not in that from saline-administrated rats (Figure 7B). By contrast, *U6 snRNA*, a small nuclear RNA ubiquitously expressed in many cell types including podocytes, was detected in urine at similar levels from both PAN- and saline-administrated rats (Figure 7B).

## 4. Discussion

This study performed miRNA-seq analysis of primary culture rat podocytes treated with or without PAN. The miRNA-seq dataset was combined with RNA-seq datasets of PAN-treated rat podocytes, which were acquired previously from the same RNA samples [25], and subjected to integrative bioinformatical analysis of miRNA and mRNA expression. *MiR-217-5p* was identified as the miRNA most strongly upregulated by PAN, with Myo1d as its possible target. We propose that the *miR-217-5p*-Myo1d axis may be associated with PAN-induced podocyte injury.

Many miRNAs have been previously associated with podocyte injury [23,24]. This study identified *miR-217-5p* as having the most highly upregulated expression (5.73-fold) following PAN-induced podocyte injury. The second most highly upregulated miRNA was *miR-216a-5p* (4.64-fold). These results are consistent with the fact that *miR-217*, *-216a*, and *-216b* are generated from a common host gene designated as *MIR217HG*, which spans an approximately 40-kbp region in the rat, mouse, and human genomes, allowing simultaneous regulation at a transcriptional level. Furthermore, the expression of *MIR217HG*-derived miRNAs is simultaneously dysregulated in different tissues under pathological conditions, such as in acute pancreatic injury [38,39,40,41,42,43], pancreatic cancer [44,45], and gastric cancers [46]; this may also be the case with podocyte injury.

Of the five dysregulated miRNAs, only *miR-217-5p* has been associated with podocyte injury in previous studies. Sun et al. reported that the expression of *miR-217-5p* is upregulated in podocytes stimulated with high glucose, and *miR-217-5p* is involved in high glucose-induced podocyte injury by attenuating the PTEN expression as its direct target [47]. These data are consistent with our findings that *miR-217-5p* expression is upregulated in response to podocyte injury. However, Jin et al. showed that reduced expression of the long noncoding RNA XIST prevents podocyte apoptosis in membranous nephropathy through the *miR-217*-TLR4 axis [48]. This report contrasts with our finding because claiming that *miR-217-5p* exerts a protective effect on podocyte injury. The inconsistency may be attributed to different pathological conditions between studies. The PAN nephropathy model exhibits pathological conditions similar to a minimal change disease rather than membranous nephropathy. However, whether *miR-217-5p* expression is differently regulated in various nephropathy models requires further investigation.

Although dysregulation of *miR-217-5p* expression to podocyte injury has been reported previously, the mechanism underlying the relationship is not understood. The GO enrichment analysis of our RNA-seq dataset showed that the predicted targets of *miR-217-5p* were closely associated with cell morphogenesis. Consistent with this analysis, E11 cells with *miR-217-5p* overexpression exhibited shrunken cell shapes with shortened or disorganized actin cytoskeletons. Previously, Solanki et al. demonstrated that the apoptosis-related p53 pathway is involved in actin cytoskeleton damage in PAN- or ADR-treated podocytes [36]. However, in our study, the p53 pathway was not associated with predicted targets of *miR-217-5p* in PAN-treated podocytes. Furthermore, unlike PAN, *miR-217-5p* overexpression did not affect E11 cell viability. Therefore, it is possible that PAN induces podocyte cell death through a mechanism independent of *miR-217-5p*. Of note, pathway analysis using DAVID (the Database for Annotation, Visualization and Integrated Discovery) revealed that mRNAs identified to be upregulated by PAN in our mRNA-seq analysis are most significantly associated with the p53 signaling pathway (Table S6). Therefore, we suggest that PAN may also induce morphological changes of podocytes as a result of p53-mediated apoptosis activated by the upregulated mRNAs. On the other hand, the mRNAs identified to be downregulated by PAN were not significantly associated with the p53 signaling pathway. Instead, these mRNAs were strongly associated with cell morphology-related pathways such as the “regulation of actin cytoskeleton” and “focal adhesion” pathways (Table S7), which were not associated with the upregulated mRNAs. It is possible that these pathways are partly associated with mRNAs negatively regulated by the PAN-induced miRNAs including *miR-217-5p*.

Interestingly, pathway analysis using DAVID showed that the predicted targets of *miR-217-5p* in podocytes are significantly associated with the ‘axon guidance’ pathway. Previous studies have suggested there is common molecular machinery for the formation of podocyte foot processes and the axon guidance of neurons [49,50,51]. Taken together with the fact that the myosin I protein family was previously associated with both podocyte morphology [52] and axon guidance [53], we decided to focus on Myo1d, an actin-binding protein, as a possible target of *miR-217-5p*. As expected, Myo1d is a direct target of *miR-217-5p*; therefore, the *miR-217-5p*-Myo1d axis could be involved in podocyte injury and warrants further investigation as a research target for clinical applications.

Finally, we showed that *miR-217-5p* was present in urine from PAN-administrated rats but not in control rats. When podocytes are severely damaged, they occasionally become detached from the glomeruli and moved into urine [54]. Furthermore, urine is rich in exosomes, unilamellar small vesicles (50–100 nm in diameter) secreted from many cell types including podocytes [54]. Consistently, we detected small vesicles with varying diameter peaking at 100 nm in the PAN- and saline-administrated rats by dynamic light scattering (DLS) (Figure S8). Previous work demonstrated that intracellular miRNAs are loaded into exosomes and secreted into the extracellular environment [55]; therefore, it is possible that *miR-217-5p* is present in not only urinary podocytes but also urinary exosomes from PAN-administrated rats. Taken together, *miR-217-5p* may be a promising biomarker of some kidney diseases involving podocyte injury.

In the present study, we could identify a novel miRNA-mRNA axis associated with podocyte injury. Nonetheless, this study has some limitations. First, it should be demonstrated that decreased Myo1d expression in E11 and rat primary podocytes leads to their morphological changes. Furthermore, the loss-of-function studies of *miR-217-5p*, e.g., the phenotypic analysis of *miR-217-5p*-knockout podocytes generated using a genome editing technique, is necessarily performed to support our findings. We hope that the results of such additional experiments will increase the clinical significance of our findings.

## Figures and Tables

**Table 1:** miRNAs dysregulated in PAN-treated podocytes.

miRNA	Fold Change	Regulation
*rno-miR-217-5p*	5.733	Upregulated
*rno-miR-216a-5p*	4.641	Upregulated
*rno-miR-338-3p*	2.295	Upregulated
*rno-miR-3583-5p*	2,125	Upregulated
*rno-miR-3572*	0.3526	Downregulated

## Data Availability

The small RNA sequencing data obtained for this study are deposited in DRA of DDBJ under the accession identifier DRA013173.

## Supplementary Materials

The following information can be downloaded at https://www.mdpi.com/article/10.3390/ncrna8030043/s1. Figure S1. Effect of induced *miR-217-5p* expression on cell viability of E11 cells. Cell viability was evaluated at day 4 after transfection. n.s.: not significant compared to the NC-transfected cells. Figure S2. Selection of mRNAs dysregulated in PAN-treated podocytes. The numbers of upregulated (A) and downregulated (B) mRNAs selected based on the criteria are shown. Figure S3. Selection of mRNAs possibly targeted by *miR-217-5p*. Figure S4. Interactive GO terms enriched with the predicted targets of *miR-217-5p* in PAN-treated podocytes. Enriched GO terms (biological process) were filtered with a threshold of *p*-values greater than 8.35 × 10^−5^ and analyzed for interconnection using the GOnet software. Of the 12 predicted targets, only Cris1 is not connected to any of the enriched GO terms. The more intense color of the GO term node indicates the more significant enrichment of the term (the smaller *p*-value). Figure S5. *Myo1d* mRNA levels in PAN-treated primary rat podocytes. Values were normalized relative to *GAPDH* mRNA expression. Primary rat podocytes were treated with 0–30 μg/mL PAN. The relative expression level of Myo1d mRNA at 0 μg/mL PAN was given an arbitrary value of 1. * *p* < 0.05 versus 0 mg/mL PAN. Data represent the means ± SD. Figure S6. Immunocytochemical detection of Myo1d protein in E11 cells. Myo1d proteins were detected in the cytosol of E11 cells. The cytosolic localization of Myo1d proteins have been also shown in the human bone osteosarcoma cell line U-2 OS (The Human Protein Atlas database; https://www.proteinatlas.org/ENSG00000176658-MYO1D/subcellular#, accessed on 6 June 2022). Bar: 100 μm. Figure S7. Western blot images showing the detection of Myo1d protein in E11 cells with induced *miR-217-5p* expression. Arrowheads indicate the immunodetected bands of Myo1d protein. Figure S8. Size distribution of particles in urine from Wistar rats administrated with PAN (100 mg/kg) or saline. Whole particles in the urine were isolated by ultracentrifugation and analyzed by dynamic light scattering. Table S1. Primers designed for SYBR qRT-PCR. Table S2. Summary of small RNA sequencing results. Table S3. mRNAs upregulated in response to PAN-induced podocyte injury. Table S4. mRNAs downregulated in response to PAN-induced podocyte injury. Table S5. Decreased *Myo1d* mRNA expression in injured human podocytes based on the RNA sequencing datasets registered under the accession GSE124622 in Gene Expression Omnibus. Table S6. Pathways significantly associated with the mRNAs upregulated by PAN in podocytes. Table S7. Pathways significantly associated with the mRNAs downregulated by PAN in podocytes.

## References

[1] (2015). World Kidney Day: Chronic Kidney Disease. http://www.worldkidneyday.org/faqs/chronic-kidney-disease/.

[2] Whaley-Connell A., Nistala R., Chaudhary K. (2011). The importance of early identification of chronic kidney disease. Mo. Med..

[3] Darshi M., Van Espen B., Sharma K. (2016). Metabolomics in Diabetic Kidney Disease: Unraveling the Biochemistry of a Silent Killer. Am. J. Nephrol..

[4] Vaidya S.R., Aeddula N.R. (2021). Chronic Renal Failure. StatPearls.

[5] Zhong J., Yang H.C., Fogo A.B. (2017). A perspective on chronic kidney disease progression. Am. J. Physiol. Renal. Physiol..

[6] Turin T.C., Tonelli M., Manns B.J., Ravani P., Ahmed S.B., Hemmelgarn B.R. (2012). Chronic kidney disease and life expectancy. Nephrol. Dial. Transplant..

[7] Ng J.K., Li P.K. (2018). Chronic kidney disease epidemic: How do we deal with it?. Nephrology (Carlton).

[8] Whalen H., Clancy M., Jardine A. (2012). Future challenges in renal transplantation. Minerva Chir..

[9] Neipp M., Jackobs S., Klempnauer J. (2009). Renal transplantation today. Langenbecks Arch. Surg..

[10] Coates P.T., Wong G. (2020). Current controversies in nephrology—how to crossmatch for transplantation?. Kidney Int..

[11] Sandip K. (2015). Challenges & Controversies in Kidney Transplantation.

[12] Shrestha B.M. (2009). Strategies for reducing the renal transplant waiting list: A review. Exp. Clin. Transplant..

[13] Voora S., Adey D.B. (2019). Management of Kidney Transplant Recipients by General Nephrologists: Core Curriculum 2019. Am. J. Kidney. Dis..

[14] Murray I., Paolini M.A. (2021). Histology, Kidney and Glomerulus. StatPearls.

[15] Liapis H., Romagnani P., Anders H.J. (2013). New insights into the pathology of podocyte loss: Mitotic catastrophe. Am. J. Pathol..

[16] Garg P. (2018). A Review of Podocyte Biology. Am. J. Nephrol..

[17] Nagata M. (2016). Podocyte injury and its consequences. Kidney Int..

[18] Wakiyama M., Takimoto K., Ohara O., Yokoyama S. (2007). Let-7 microRNAmediated mRNA deadenylation and translational repression in a mammalian cell-free system. Genes Dev..

[19] Meister G., Tuschl T. (2004). Mechanisms of gene silencing by double-stranded RNA. Nature.

[20] Shi S., Yu L., Chiu C., Sun Y., Chen J., Khitrov G., Merkenschlager M., Holzman L.B., Zhang W., Mundel P. (2008). Podocyte-selective deletion of dicer induces proteinuria and glomerulosclerosis. J. Am. Soc. Nephrol..

[21] Harvey S.J., Jarad G., Cunningham J., Goldberg S., Schermer B., Harfe B.D., McManus M.T., Benzing T., Miner J.H. (2008). Podocyte-specific deletion of dicer alters cytoskeletal dynamics and causes glomerular disease. J. Am. Soc. Nephrol..

[22] Ho J., Ng K.H., Rosen S., Dostal A., Gregory R.I., Kreidberg J.A. (2008). Podocyte-specific loss of functional microRNAs leads to rapid glomerular and tubular injury. J. Am. Soc. Nephrol..

[23] Ishii H., Kaneko S., Yanai K., Aomatsu A., Hirai K., Ookawara S., Ishibashi K., Morishita Y. (2020). MicroRNAs in Podocyte Injury in Diabetic Nephropathy. Front. Genet..

[24] Li J.Y., Yong T.Y., Michael M.Z., Gleadle J.M. (2010). Review: The role of microRNAs in kidney disease. Nephrology (Carlton).

[25] Horikawa A., Yoneda T., Yaoita E., Yamaguchi K., Shigenobu S., Kuramochi M., Yamate J., Inui T., Ishibashi O. (2019). A novel splicing variant of small nucleolar RNA host gene 4 is a podocyte-selective non-coding RNA upregulated in response to puromycin aminonucleoside-induced podocyte injury. J. Biochem..

[26] Katsuya K., Yaoita E., Yoshida Y., Yamamoto Y., Yamamoto T. (2006). An improved method for primary culture of rat podocytes. Kidney Int..

[27] Saito T., Yamada E., Okada S., Shimoda Y., Tagaya Y., Hashimoto K., Satoh T., Mori M., Okada J., Pessin J.E. (2014). Nucleobindin-2 is a positive regulator for insulin-stimulated glucose transporter 4 translocation in fenofibrate treated E11 podocytes. Endocr. J..

[28] Ishibashi O., Akagi I., Ogawa Y., Inui T. (2018). *MiR-141-3p* is upregulated in esophageal squamous cell carcinoma and targets pleckstrin homology domain leucine-rich repeat protein phosphatase-2, a negative regulator of the PI3K/AKT pathway. Biochem. Biophys. Res. Commun..

[29] Pomaznoy M., Ha B., Peters B. (2018). GOnet: A tool for interactive Gene Ontology analysis. BMC Bioinform..

[30] Thilo F., Liu Y., Loddenkemper C., Schuelein R., Schmidt A., Yan Z., Zhu Z., Zakrzewicz A., Gollasch M., Tepel M. (2012). VEGF regulates TRPC6 channels in podocytes. Nephrol. Dial. Transplant..

[31] Nosaka K., Takahashi T., Nishi T., Imaki H., Suzuki T., Suzuki K., Kurokawa K., Endou H. (1997). An adenosine deaminase inhibitor prevents puromycin aminonucleoside nephrotoxicity. Free Radic. Biol. Med..

[32] Xia L., Zhou M., Kalhorn T.F., Ho H.T., Wang J. (2009). Podocyte-specific expression of organic cation transporter PMAT: Implication in puromycin aminonucleoside nephrotoxicity. Am. J. Physiol. Renal. Physiol..

[33] Guo H., Ingolia N.T., Weissman J.S., Bartel D.P. (2010). Mammalian microRNAs predominantly act to decrease target mRNA levels. Nature.

[34] Fabian M.R., Sonenberg N., Filipowicz W. (2010). Regulation of mRNA translation and stability by microRNAs. Annu. Rev. Biochem..

[35] Noris M., Remuzzi G. (2012). Non-muscle myosins and the podocyte. Clin. Kidney J..

[36] Solanki A.K., Srivastava P., Rahman B., Lipschutz J.H., Nihalani D., Arif E. (2019). The Use of High-throughput transcriptomics to identify pathways with therapeutic significance in podocytes. Int. J. Mol. Sci..

[37] Okabe M., Motojima M., Miyazaki Y., Pastan I., Yokoo T., Matsusaka T. (2019). Global polysome analysis of normal and injured podocytes. Am. J. Physiol. Renal. Physiol..

[38] Erdos Z., Barnum J.E., Wang E., DeMaula C., Dey P.M., Forest T., Bailey W.J., Glaab W.E. (2020). Evaluation of the Relative Performance of Pancreas-Specific MicroRNAs in Rat Plasma as Biomarkers of Pancreas Injury. Toxicol. Sci..

[39] Li Z., Rouse R. (2018). Co-sequencing and novel delayed anti-correlation identify function for pancreatic enriched microRNA biomarkers in a rat model of acute pancreatic injury. BMC Genom..

[40] Wang J., Huang W., Thibault S., Brown T.P., Bobrowski W., Gukasyan H.J., Evering W., Hu W., John-Baptiste A., Vitsky A. (2017). Evaluation of *miR-216a* and *miR-217* as Potential Biomarkers of Acute Exocrine Pancreatic Toxicity in Rats. Toxicol. Pathol..

[41] Rouse R., Rosenzweig B., Shea K., Knapton A., Stewart S., Xu L., Chockalingam A., Zadrozny L., Thompson K. (2017). MicroRNA biomarkers of pancreatic injury in a canine model. Exp. Toxicol. Pathol..

[42] Calvano J., Edwards G., Hixson C., Burr H., Mangipudy R., Tirmenstein M. (2016). Serum microRNAs-217 and -375 as biomarkers of acute pancreatic injury in rats. Toxicology.

[43] Goodwin D., Rosenzweig B., Zhang J., Xu L., Stewart S., Thompson K., Rouse R. (2014). Evaluation of *miR-216a* and *miR-217* as potential biomarkers of acute pancreatic injury in rats and mice. Biomarkers.

[44] Rachagani S., Macha M.A., Menning M.S., Dey P., Pai P., Smith L.M., Mo Y.Y., Batra S.K. (2015). Changes in microRNA (miRNA) expression during pancreatic cancer development and progression in a genetically engineered KrasG12D; Pdx1-Cre mouse (KC) model. Oncotarget.

[45] Egeli U., Tezcan G., Cecener G., Tunca B., Demirdogen Sevinc E., Kaya E., Ak S., Dundar H.Z., Sarkut P., Ugras N. (2016). Pancreas. *miR-216b* Targets FGFR1 and Confers Sensitivity to Radiotherapy in Pancreatic Ductal Adenocarcinoma Patients Without EGFR or KRAS Mutation. Pancreas.

[46] Safaralizadeh R., Ajami N., Nemati M., Hosseinpourfeizi M., Azimzadeh Isfanjani A., Moaddab S.Y. (2019). Disregulation of *miR-216a* and *miR-217* in Gastric Cancer and Their Clinical Significance. J. Gastrointest. Cancer.

[47] Sun J., Li Z.P., Zhang R.Q., Zhang H.M. (2017). Repression of *miR-217* protects against high glucose-induced podocyte injury and insulin resistance by restoring PTEN-mediated autophagy pathway. Biochem. Biophys. Res. Commun..

[48] Jin L.W., Pan M., Ye H.Y., Zheng Y., Chen Y., Huang W.W., Xu X.Y., Zheng S.B. (2019). Down-regulation of the long non-coding RNA XIST ameliorates podocyte apoptosis in membranous nephropathy via the *miR-217*-TLR4 pathway. Exp. Physiol..

[49] Kobayashi N. (2002). Mechanism of the process formation; podocytes vs. neurons. Microsc. Res. Tech..

[50] Tapia R., Guan F., Gershin I., Teichman J., Villegas G., Tufro A. (2008). Semaphorin3a disrupts podocyte foot processes causing acute proteinuria. Kidney Int..

[51] Fan X., Li Q., Pisarek-Horowitz A., Rasouly H.M., Wang X., Bonegio R.G., Wang H., McLaughlin M., Mangos S., Kalluri R. (2012). Inhibitory effects of Robo2 on nephrin: A crosstalk between positive and negative signals regulating podocyte structure. Cell Rep..

[52] Arif E., Wagner M.C., Johnstone D.B., Wong H.N., George B., Pruthi P.A., Lazzara M.J., Nihalani D. (2011). Motor protein Myo1c is a podocyte protein that facilitates the transport of slit diaphragm protein Neph1 to the podocyte membrane. Mol. Cell Biol..

[53] Benesh A.E., Fleming J.T., Chiang C., Carter B.D., Tyska M.J. (2012). Expression and localization of myosin-1d in the developing nervous system. Brain Res..

[54] Zeng L., Szeto C.C. (2021). Urinary podocyte markers in kidney diseases. Clin. Chim. Acta.

[55] Valadi H., Ekström K., Bossios A., Sjöstrand M., Lee J.J., Lötvall J.O. (2007). Exosome-mediated transfer of mRNAs and microRNAs is a novel mechanism of genetic exchange between cells. Nat. Cell Biol..

