# Supplementary material for: The Role of miR-217-5p in the Puromycin Aminonucleoside-Induced Morphological Change of Podocytes
Source: Noncoding RNA. 2022 Jun 8;8(3):43. doi: 10.3390/ncrna8030043 (PMC9229466; doi:10.3390/ncrna8030043)
Supplement: Supplementary file 1 [file ncrna-08-00043-s001.zip › SupplFigure_revise_ver6.pdf]

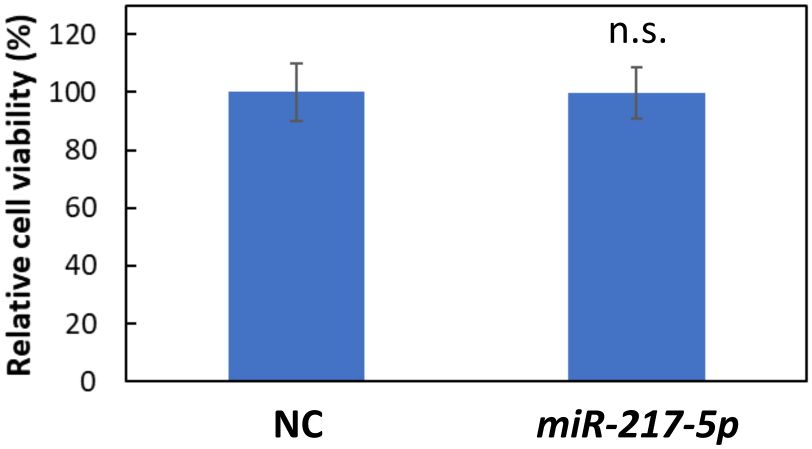

Figure S1. Effect of induced *miR-217-5p* expression on cell viability of E11 cells. Cell viability was evaluated at day 4 after transfection. n.s.: not significant compared to the NC-transfected cells.

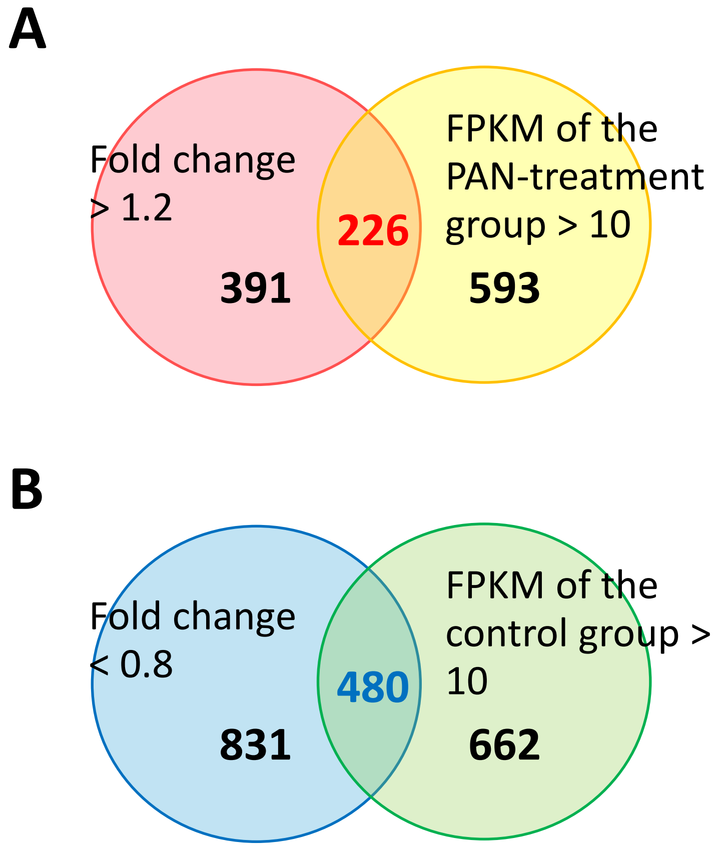

Figure S2. Selection of mRNAs dysregulated in PAN-treated podocytes. The numbers of upregulated (A) and downregulated (B) mRNAs selected based on the criteria are shown.

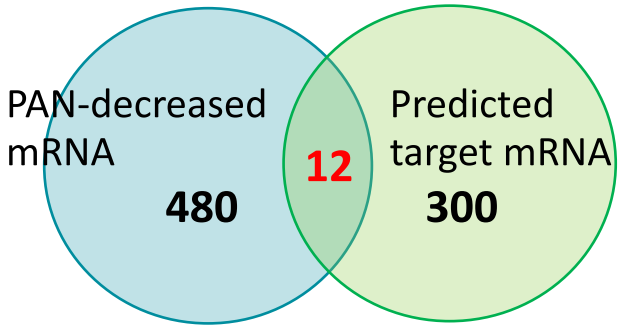

Figure S3. Selection of mRNAs possibly targeted by *miR-217-5p.*

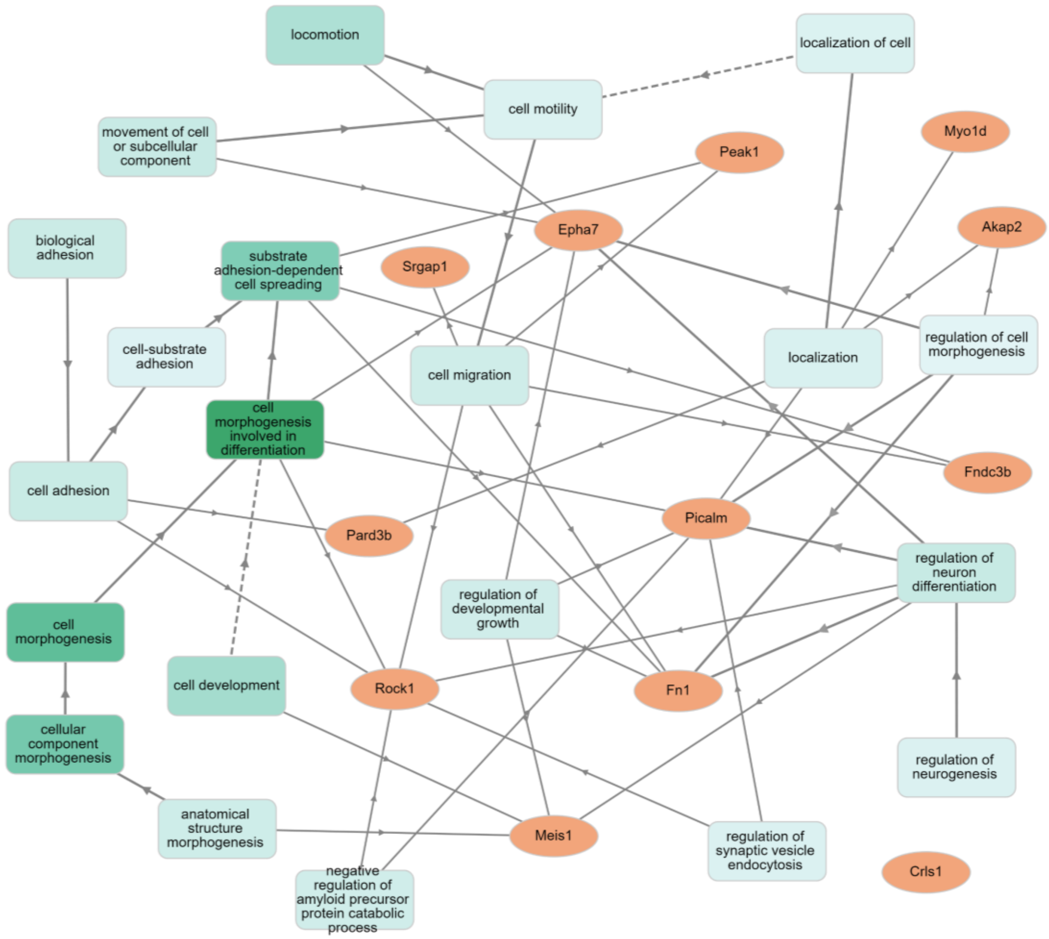

Figure S4. Interactive GO terms enriched with the predicted targets of *miR-217-5p* in PAN-treated podocytes. Enriched GO terms (biological process) were filtered with a threshold of *p*-values greater than 8.35 x 10^{-5} and analyzed for interconnection using the GOnet software. Of the 12 predicted targets, only Cris1 is not connected to any of the enriched GO terms. The more intense color of the GO term node indicates the more significant enrichment of the term (the smaller *p*-value).

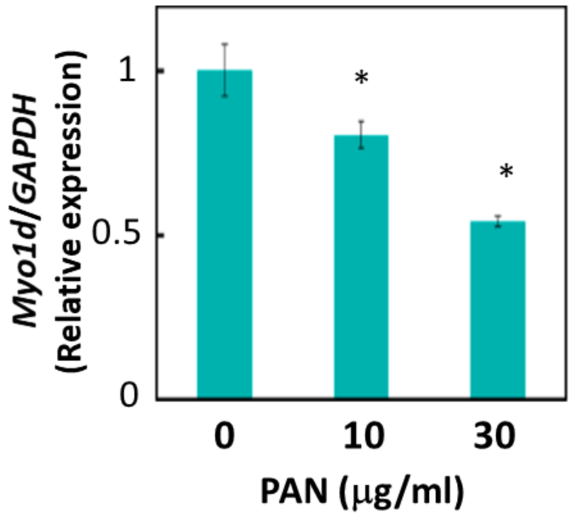

Figure S5. *Myo1d* mRNA levels in PAN-treated primary rat podocytes. Values were normalised relative to *GAPDH* mRNA expression. Primary rat podocytes were treated with 0-30 μg/ml PAN. The relative expression level of Myo1d mRNA at 0 μg/ml PAN was given an arbitrary value of 1. *p < 0.05 versus 0 μg/ml PAN. Data represent the means ± SD.

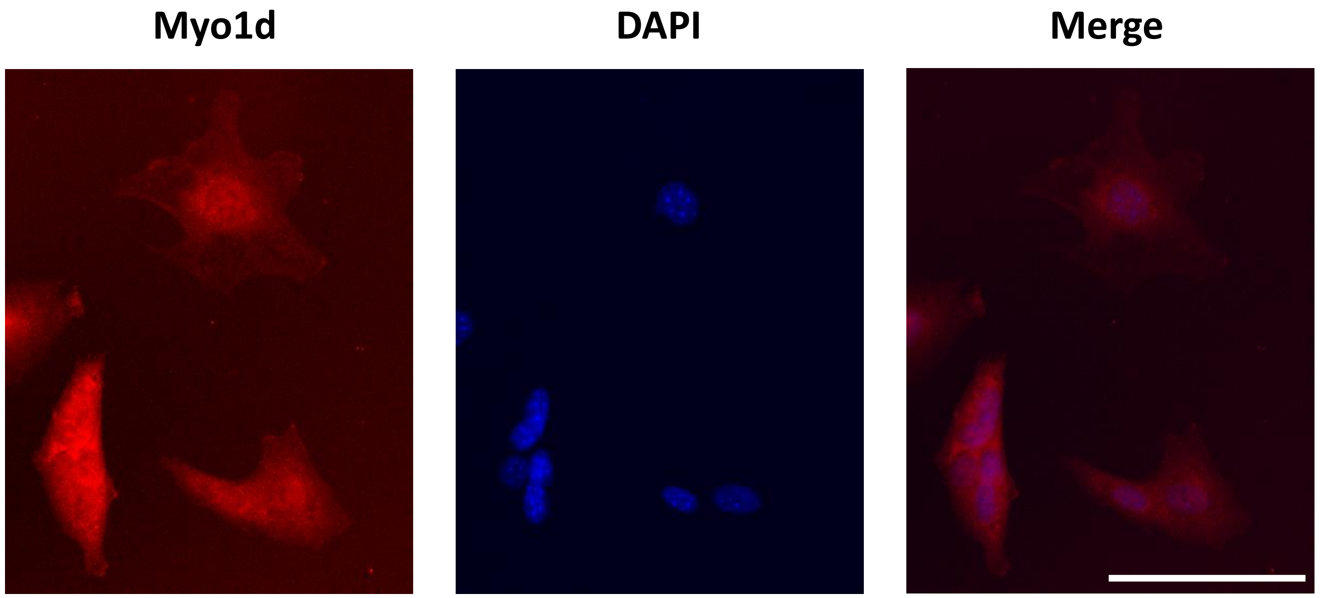

Figure S6. Immunocytochemical detection of Myo1d protein in E11 cells. Myo1d proteins were detected in the cytosol of E11 cells. The cytosolic localisation of Myo1d proteins have been also shown in the human bone osteosarcoma cell line U-2 OS (The Human Protein Atlas database; https://www.proteinatlas.org/ENSG00000176658-MYO1D/subcellular#). Bar: 100 μm.

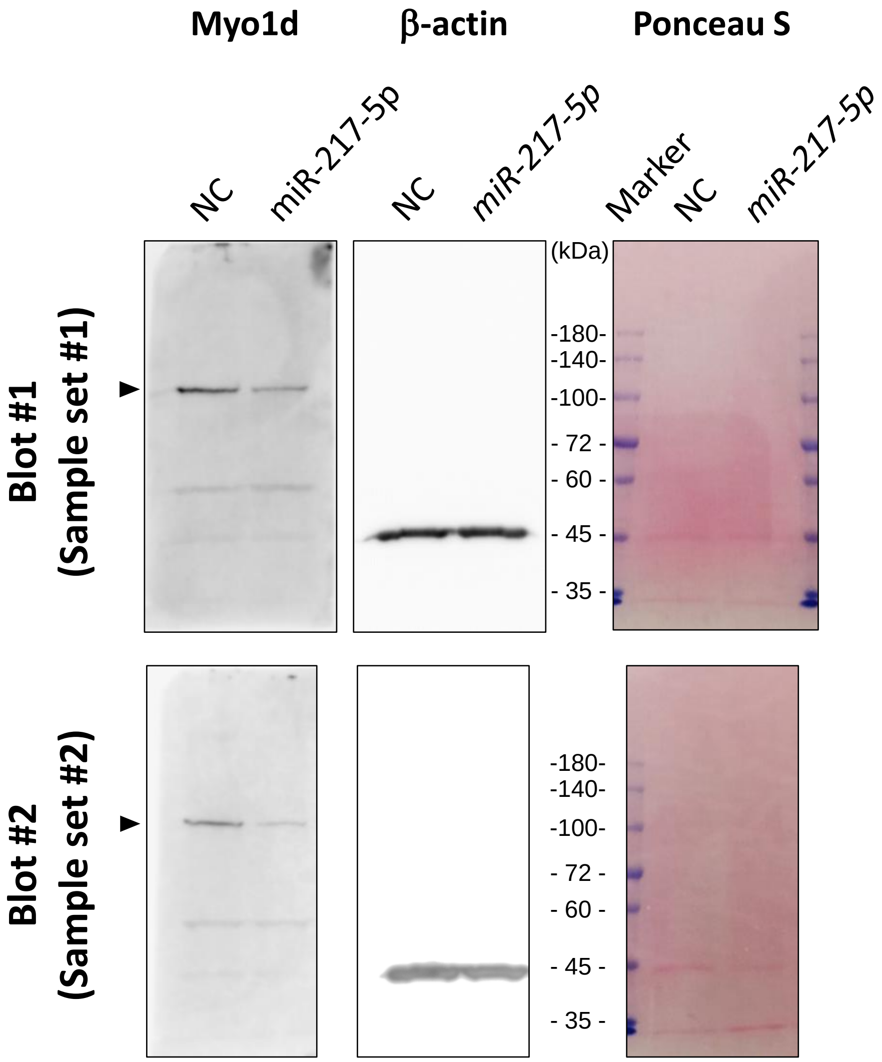

Figure S7. Western blot images showing the detection of Myo1d protein in E11 cells with induced *miR-217-5p* expression. Arrowheads indicate the immunodetected bands of Myo1d protein.

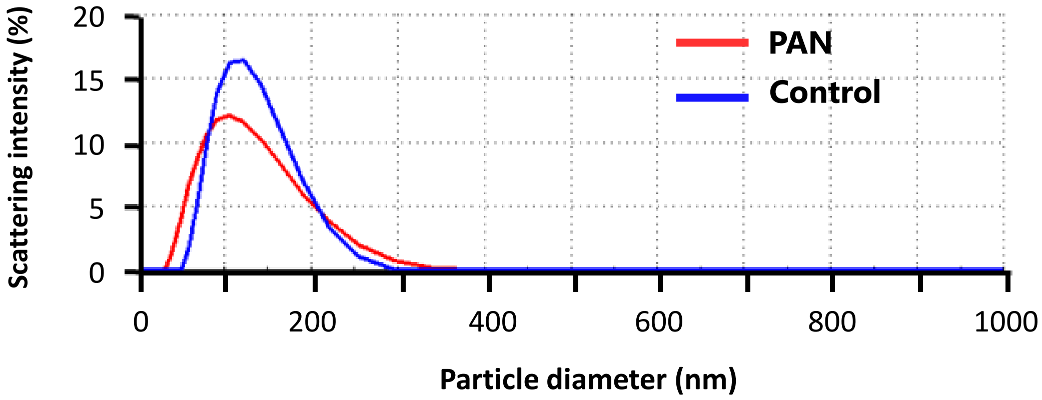

Figure S8. Size distribution of particles in urine from Wistar rats administrated with PAN (100 mg/kg) or saline. Whole particles in the urine were isolated by ultracentrifugation and analysed by dynamic light scattering.
