# Supplementary material for: The Role of miR-217-5p in the Puromycin Aminonucleoside-Induced Morphological Change of Podocytes
Source: Noncoding RNA. 2022 Jun 8;8(3):43. doi: 10.3390/ncrna8030043 (PMC9229466; doi:10.3390/ncrna8030043)
Supplement: Supplementary file 1 [file ncrna-08-00043-s001.zip › Table S1_MinRevise.pdf]

Table S1. Primers designed for SYBR qRT-PCR.

Target		Sequence (5’ to 3’)
Mouse Myo1d	Forward	GAGAGCCTTGAGTTCGGCAA
	Reverse	GTCCTTTCTCAAATCTGAGCCT
Rat Myo1d	Forward	GTCATGTCCGAAAGGTGAATCG
	Reverse	GACACACTCAGGCCAGTCAA
Rat GAPDH	Forward	GGACCTGGACGACATAGTGC
	Reverse	CCGCCAACATGATTCTCCGA
Mouse & rat 18S rRNA	Forward	GGCCCTGTAATTGGAATGAGTC
	Reverse	CCAAGATCCAACTACGAGCTT
