# Supplementary material for: The Role of miR-217-5p in the Puromycin Aminonucleoside-Induced Morphological Change of Podocytes
Source: Noncoding RNA. 2022 Jun 8;8(3):43. doi: 10.3390/ncrna8030043 (PMC9229466; doi:10.3390/ncrna8030043)
Supplement: Supplementary file 1 [file ncrna-08-00043-s001.zip › Table_S2_MinRevise.pdf]

Table S2. The summary of small RNA sequencing results.

	podocyte_1_cont_R1 (absolute values)	podocyte_1_PAN_R1 (absolute values)	podocyte_2_cont_R1 (absolute values)	podocyte_2_PAN_R1 (absolute values)	podocyte_3_cont_R1 (absolute values)	podocyte_3_PAN_R1 (absolute values)
Total number of reads	12,868,529	13,350,898	12,094,850	13,004,992	8,635,242	13,479,157
Aligned reads	11,110,540	12,109,253	10,342,259	11,533,336	7,624,667	11,132,414
- Uniquely matched reads	7,018,093	8,113,886	6,572,343	7,517,159	4,917,024	6,576,953
- Multiply matched reads	2,528,594	2,578,279	2,186,365	2,540,933	1,625,248	2,735,021
- Partially aligned reads	1,563,853	1,417,088	1,583,551	1,475,244	1,082,395	1,820,440
Unaligned reads	1,367,472	1,002,074	1,455,526	1,265,173	789,600	1,832,455
- No matches found	225,239	93,971	403,801	166,271	181,952	241,442
- Too many matches	1,142,233	908,103	1,051,725	1,098,902	607,648	1,591,013
Reads ignored due to failure of vendor QC	0	0	0	0	0	0
Reads ignored due to small size	390,517	239,571	297,065	206,483	220,975	514,288
Maximum read length	50	50	50	50	50	50
Average read length	19	19	21	20	21	19
