# Supplementary material for: The Role of miR-217-5p in the Puromycin Aminonucleoside-Induced Morphological Change of Podocytes
Source: Noncoding RNA. 2022 Jun 8;8(3):43. doi: 10.3390/ncrna8030043 (PMC9229466; doi:10.3390/ncrna8030043)
Supplement: Supplementary file 1 [file ncrna-08-00043-s001.zip › Table_S5_MinRevise.pdf]

Table S5. Decreased *Myo1d* mRNA expression in injured human podocytes based on the RNA sequencing datasets registered under the accession GSE124622 in Gene Expression Omnibus.

Podocyte injury inducer	RPKM[a] (Control)			RPKM (Experiment)			Basement mean	Fold change	*P* value	*P* adj[b]
	No. 1	No. 2	No. 3	No. 1	No. 2	No. 3				
PAN	638	1023	1456	482	394	487	744	0.410	7.55E-06	2.86E-05
Adriamycin	638	1023	1456	422	706	602	779	0.641	0.0364	0.118

[a]RPKM: reads per kilobase of exon per million mapped reads

[b]*P* value adjusted using Benjamini & Hochberg method.
